# Special Issue “Phytohormones: Important Participators in Plant Growth and Development”

**DOI:** 10.3390/ijms25031380

**Published:** 2024-01-23

**Authors:** Guzel Kudoyarova

**Affiliations:** Ufa Institute of Biology, Ufa Federal Research Centre of the Russian Academy of Sciences, Pr. Octyabrya, 69, 450054 Ufa, Russia; guzel@anrb.ru

The articles published in the *IJMS* Special Issue “Phytohormones” are devoted to various aspects of hormonal control of plant growth and development promoting adaptation to normal and stress conditions. They report on findings related to key stages of the plant life cycle such as flower opening and closure [1], development of inflorescence and kernel meristem [2], fruit ripening and senescence [3] and seed maturation [4]. Important developmental processes like shoot branching [5], deposition of lignin and suberin and the formation of apoplastic barriers [6] are the topics of some papers. Several articles address the involvement of classical hormones (cytokinins [6,7], auxins [8,9,10], abscisic acid and gibberellins [4]) in the regulation of plants growth and development. This collection also contains reports on the action of plant growth regulators recently classified as phytohormones: strigalactones [5] and melatonin [3,7]. Hormonal control of plant growth and development is discussed in connection with the development of plant resistance to stress conditions [5,7,11]. The novelty of the collection presented in this SI lies in demonstrating new aspects of hormonal control of physiological processes: the disturbance of hormonal balance in a maize mutant characterized by unusually large embryo [2], the involvement of ABA in the control of the movement of water lily flower petals [1], melatonin-induced increase in cherimoya fruit resistance to chilling [3], hormonal control of accumulation of storage proteins in Yellow lupin [4], identification of genes responsible for the synthesis of strigolactones in *Chimonanthus praecox* and their involvement in shoot branching [5], discovery of cytokinin ability to slow down the formation of Casparian bands in transgenic tobacco plants [6] and interaction of cytokinins with melatonin in the control of plant resistance to high light stress [7], identification of the YUCCA genes in *Mikania micrantha* [8], regulation of PIN-formed (PIN) auxin transporters by their degradation [9], interaction of hormonal and electrical signals in plants [11]. The importance of genes related to abscisic acid (ABA) and modifications of cell walls in controlling the opening and closing of water lily flowers is demonstrated in the paper by Zhou et al. published in the present Special Issue [1]. It is suggested that ABA-induced flowering is due to the interaction of this phytohormone with the pathways related to cell wall modifications. The opening and closing movement of petals is influenced by many external and internal factors, including hormones that play an important role in the control of these processes [12]. For example, it was shown that auxin controls circadian flower opening and closure in water lilies [13], while ethylene regulates asymmetric growth of the petal base thereby promoting flower opening in roses [14]. ABA was shown to be involved in the control of flowering time [15]. However, the mechanism of flower opening and closing by ABA has not received sufficient attention. Cell wall modifications are essential for petal development and movement [16], and ABA is able to regulate this process [17], as confirmed by the report of Zhou et al. [1].

The article by Wang et al. [2] presents the results of a study of genes involved in the control of the development of inflorescence and kernel meristem of reversed kernel1 (rk1) maize mutant characterized by an unusually large embryo. The developmental activity of inflorescence directly affects the reproductive potential of plants. Phytohormones have extremely important regulatory effects on inflorescence development. Efflux-dependent auxin gradients are responsible for plant organ formation [18], including inflorescence promoridia [19]. YUCCA proteins, which are involved in auxin biosynthesis, function in floral organ primordia [20]. Floret differentiation is inhibited unless optimal levels of gibberrelic acid (GA) are achieved [21]. In addition, it was shown that cytokinin accumulation during maize floret development inhibits the abortion of inferior floret pistils during ear differentiation [22]. ABA affects the fertility of florets during maize ear differentiation, thereby regulating the number of kernels in the ear [23]. A study of mutants in genes that control jasmonic acid (JA) synthesis indicates the involvement of this hormone in sex determination in maize [24]. A transcriptome analysis and hormone assay carried out by Wang et al. [2] revealed the importance of maintaining hormonal balance for proper florescence development and its disturbance in *krl* mutant.

Medina-Santamarina and co-authors [3] showed that treating cherimoya fruits with melatonin increased chilling tolerance by reducing chilling-induced postharvest ripening and senescence and increasing fruit quality. The protective effects of melatonin were associated with a decrease in ion leakage and chlorophyll degradation, as well as activation of antioxidant systems, which was due to a delay in ethylene production. Cold storage is the most used method to preserve fruit quality. However, tropical and subtropical fruit species, including cherimoya, are susceptible to chilling injury (CI) with symptoms such as abnormal ripening and browning [25,26]. Melatonin is an endogenous indole compound with multiple biological functions in plants [27,28,29], including plant development, stress resistance, fruit growth and ripening. Medina-Santamarina and co-authors assumed and demonstrated that melatonin treatment increases chilling resistance of cherimoya.

The article by Klajn et al. [4] addresses the involvement of hormones in controlling the accumulation of storage proteins (conglutins) in Yellow lupin. The authors emphasize the importance of plant storage proteins in terms of protein deficiency in the world population’s nutrition. The effects of abscisic acid and gibberellins on the expression of genes involved in the control of conglutins have been studied and discussed [4]. The involvement of phytohormones in the control of seed filling is considered very important [30,31]. The most significant role is attributed to the interactions between GA and ABA [32,33]. However, as numerous studies have shown, ABA especially may interact with ethylene, jasmonic acid or brassinosteroids [32,33,34,35]. The article by Klajn et al. is a significant contribution to solving the problem of hormonal control of accumulation of storage proteins in the developing seeds.

Genes encoding Cytochrome P450 Monooxygenase and responsible for the synthesis of strigolactones [36] have been identified in *Chimonanthus praecox* by Zhang and co-authors [5], and the expression of the genes was detected in the roots and shoot tips of the plants. It was shown that expression of the gene in Arabidopsis affected shoot branching [5], which confirmed involvement of strigolactone synthesis in the control of this process suggested previously [36,37].

Vysotskaya et al. [6] present the results of a study on the effect of cytokinin accumulation induced in transgenic tobacco plants, regarding the deposition of suberin and lignin and the formation of apoplastic barriers. Cytokinins are known to keep stomata open [38,39,40]. However, this effect of cytokinins can be detrimental unless elevated transpiration is balanced by an increase in plant hydraulic conductance, which is known to depend on transcellular transport through aquaporins (AQPs) [41] and the formation of apoplastic barriers [42,43]. The article by Vysotskaya et al. was the first to show that cytokinins slow down the formation of Casparian bands in roots and that hydraulic conductivity in plants is additionally maintained by increasing the activity of water channels, aquaporins.

The topic of control of plant tolerance by melatonin is continued in the article by Bychkov and co-authors [7], which describes the interaction of cytokinins with melatonin in the control of plant resistance to high light stress. It is suggested that a melatonin-induced reduction in the photo-damage of Arabidopsis plants can be partially explained by the effects of melatonin on the expression of the genes responsible for cytokinin metabolism and signaling. Cytokinins, in turn, affect plant resistance to high light stress due to their effects on the expression of genes involved in melatonin synthesis. The article by Bychkov et al. [7] describes the effects of high light stress and melatonin on the genes encoding isopentenyltransferase, which catalyzes the transfer of the isopentenyl group to adenine nucleotide, thereby producing cytokinin (isopentenyladenine) [44,45], LOG gene encoding cytokinin riboside 5′-monophosphate phosphoribohydrolase, which releases free cytokinin base from its nucleotide in one step [46], and other genes involved in cytokinin metabolism and signaling [47]. The authors of [7] consider cytokinins to be contributors to stress tolerance [48,49,50], especially to light stress [51].

Luo and co-authors [8] identified and studied genes from the YUCCA family in Mikania micrantha encoding tryptophan aminotransferases which catalyzed the oxidative decarboxylation of the precursor of indole-3-acetic acid to form IAA [52]. The authors studied the expression of the gene in different organs of *Mikania micrantha*. Using an IAA-sensitive reporter, they also discovered increased level of auxins in Arabidopsis transgenic plants over-expressing the *Mikania micrantha* YUCCA genes. Auxin is one of the main phytohormones that regulates plant development processes in a dose-dependent manner depending on the concentration of auxin in plant tissues [53,54,55]. Therefore, the control of auxin metabolic pathways in plant cells is most important for proper development and response to environmental stimuli [56,57]. Production of IAA catalyzed by the YUCCA (YUC) family of flavin monooxygenases (FMO) is the irreversible rate-limiting reaction for IAA biosynthesis [58]. The novelty of the research performed by Luo and co-authors is in the discovery of the YUCCA genes in *Mikania micrantha* (a widespread weed in the tropics that grows very quickly).

The article by Zhang et al. [9] addresses an important and insufficiently studied problem of regulating PIN-formed (PIN) auxin transporters by their degradation. The article highlights the importance of targeting PIN proteins to vacuoles, their ubiquitination and autophagy, as well as the involvement of cytoskeleton and plant hormones in the processes. PIN-formed (PIN) proteins are auxin efflux carriers that are extremely important for auxin-triggered organogenesis in plants [59]. The establishment of auxin gradients involves the delivery of new synthesized PIN proteins to the plasmalemma and their removal from cell membranes [60,61,62]. Recent reports highlight the roles of PIN degradation in the control of polar auxin transport [63,64], and the present study [9] deepens the understanding of its mechanism.

The theme of auxins is continued in the review by Zhang and co-authors [10]. It summarizes data on auxin signaling split into three aspects: biosynthesis and metabolism [65,66], directional transport [67] and cell/tissue-specific responses [68,69]. Although significant progress has been made in studying the diverse responses to auxin, a panoramic view of this issue is lacking and is presented in this review [10].

The final article in this collection addresses a very interesting and rarely discussed topic: the interaction of hormonal and electrical signals in plants and their importance in controlling stress responses at the whole plant level [11]. A hydraulic signal is a wave of increased hydraulic pressure, which rapidly propagates through xylem vessels, with the speed of propagation reaching tens of cm/s [70,71]. Electrical signals (ESs) are transient changes in the membrane potential, which quite rapidly propagate through tissues of the plant. The propagation speed of ESs in plants ranges from a few mm to several cm per second and depends on the type of signal and plant species [71,72,73,74]. This report [11] confirms the assumption that signals about the impact of the environment are transmitted not through one path, but through a combination of them, reflecting the nature of the stressor and its intensity.

This collection is a significant contribution to deepening knowledge about the mechanisms of hormonal action in plants.

## Data Availability

Data are contained within the article.
